# *In silico* analysis of suitable signal peptides for secretion of a recombinant alcohol dehydrogenase with a key role in atorvastatin enzymatic synthesis

**DOI:** 10.22099/mbrc.2019.31801.1372

**Published:** 2019-03

**Authors:** Mortaza Taheri-Anganeh, Seyyed Hossein Khatami, Zeinab Jamali, Amir Savardashtaki, Younes Ghasemi, Zohreh Mostafavi-Pour

**Affiliations:** 1 Department of Medical Biotechnology, School of Advanced Medical Sciences and Technologies, Shiraz University of Medical Sciences, Shiraz, Iran; 2 Recombinant Proteins Laboratory, Department of Biochemistry, School of Medicine, Shiraz University of Medical Sciences, Shiraz, Iran; 3 Cardiovascular Research Center, Shiraz University of Medical Sciences, Shiraz, Iran; 4 Department of Pharmaceutical Biotechnology, School of Pharmacy, Shiraz University of Medical Sciences, Shiraz, Iran; 5 Pharmaceutical Sciences Research Center, Shiraz University of Medical Sciences, Shiraz, Iran; 6 Maternal-Fetal Medicine Research Center, Shiraz University of Medical Sciences, Shiraz, Iran

**Keywords:** Atorvastatin, Alcohol dehydrogenase, Signal peptide, In silico

## Abstract

An elevated cholesterol level might lead to cardiovascular disease (CVD). Statins block the cholesterol synthesis pathway in the liver. Atorvastatin is the most widespread statin worldwide and, its chemical synthesis requires toxic catalysts, resulting in environmental pollution. Hence, enzymatic synthesis of atorvastatin is desirable. This process could be done by *Lactobacillus kefir* alcohol dehydrogenase (LKADH). Therefore, recombinant enzyme secretion by *Escherichia coli* using signal peptides (SPs) might result in easy production and purification. To achieve this objective, we used some online bioinformatics web servers to evaluate the suitable SPs for translocation of LKADH into extracellular spaces. “Signal Peptide Website” and “UniProt” were utilized to retrieve the SPs and LKADH sequences. “SignalP 4.1” was used to determine SPs and their cleavage site location and the results were rechecked by “Philius”. Physicochemical features of SPs were evaluated by “ProtParam”, then solubility of their fusion with LKADH was assessed by “Protein-sol”. Finally, secretion pathway and sub-cellular localization of the selected stable and soluble LKADH fusions were predicted by “PRED-TAT” and “ProtCompB”. Amongst the 41 evaluated SPs, only LPTA_ECOLI, SUBF_BACSU, CHIS_BACSU, SACB_BACAM, CDGT_BACST and AMY_BACLI could translocate LKADH out of cytoplasm. The six selected SPs in the result section were suitable to design a soluble secretory LKADH that accelerate its scale-up production and might be useful in future experimental researches.

## INTRODUCTION

Cardiovascular disease (CVD) is one of the major cause of morbidity and mortality worldwide [1]. Increased level of low-density lipoprotein cholesterol (LDL-C) is considered as the main risk factor for CVD. 3-hydroxy-3-methylglutaryl-coenzyme A (HMG-CoA) reductase inhibitors (‘statins’) were introduced since the late 1980s to prevent cardiovascular disease. Statins are potent inhibitors for HMG-CoA reductase, which block the cholesterol synthesis pathway in the liver and reduce major cardiovascular events [2]. It is proven that statins might interfere with other biological pathways as well as having several potential therapeutic effects [3]. Atorvastatin is a type of statin that reduces LDL cholesterol, which results in mortality rate reduction due to coronary heart diseases. 

Lipitor® (atorvastatin calcium) is one of the best-selling drugs in the world. The side chain of atorvastatin has two chiral cores that their synthesis is a critical step in atorvastatin synthesis process [4,5]. It was revealed that the chemical synthesis of atorvastatin needs expensive catalysts, causing extreme environmental pollutions. Therefore, applicable enzymes can reduce costs and prevent toxicity in the environment. *Lactobacillus kefir* alcohol dehydrogenase (LKADH) was introduced in 1990 by Hummel *et al*. LKADH is shown to be a beneficial enzyme for the industrial production of atorvastatin since it can act as a suitable enzyme for synthesizing the side chain of atorvastatin by reducing tert-butyl 6-chloro-3, 5-dioxohexanoate to tert-Butyl (S)-6-chloro-5-hydroxy-3-oxohexanoate, using nicotinamide adenine dinucleotide phosphate (NADPH) in a fed-batch system. NADPH is an expensive reagent, which is the limiting factor in the process of atorvastatin side chain production by LKADH; hence, its regeneration has significant importance, especially in industrial scales. On the other hand, LKADH as a single-enzyme system can effectively regenerate NADPH using cost-efficient solvent like ethanol along with the synthesis of the product. This binary function is considered as a significant advantage [6,7]. 

Recombinant DNA technology can help the industrial production of proteins by reducing the cost and increasing the efficacy of the bioprocesses. Escherichia coli (E.coli) is one of the most widespread expression hosts that can produce heterologous recombinant proteins [8]. High-level expression of recombinant proteins in *E.coli* can result in high amount aggregation of insoluble misfolded proteins in the cytoplasm, which is considered as the inclusion bodies. These aggregated intermediates are unable to get a suitable biological activity. Hence, the inclusion bodies has to be refolded to get the appropriate soluble proteins. [9]. One solution is to synthesize secretory recombinant proteins, which excrete into the periplasmic space or culture medium. Preventing protease attack, facilitated purification, correct formation of disulfide bonds and accurate protein folding are the advantages of the secretory production of recombinant proteins in comparison with cytoplasmic expression [10]. 

Considering the secretory production of recombinant proteins, they can be guided to the periplasmic space or culture medium by fusing suitable signal sequences to their N-terminus. There are several common translocation systems in the *E.coli* including Sec system, signal recognition particle-dependent (SRP-dependent) pathway, and twin-arginine translocation (TAT) system. Hence, it is possible to increase the efficiency of a translocation system using alternative signal peptides (SPs) that might be obtained from some heterologous species. A significant increase in the protein production at a commercial level, is the result of using SPs. A SP has various motifs, necessary to target a specific protein in the extra-cytoplasmic spaces. It is located at N-terminus of immature desired protein and can be detached by signal (leader) peptidase. The length of SPs is usually 15-30 amino acids that includes three distinct regions. Generally n-region consists of 5-8 positively charged residues, h-region is composed of 8-12 hydrophobic residues and, c-region contains 5-7 polar residues, which include cleavage site location in carboxyl terminus [11]. 

Various computational approaches were applied to predict a suitable N-terminal SPs, and different bioinformatics tools were utilized to predict the SPs presence and their locations [12]. In the present study, several online web servers were used to investigate suitable SPs for secretory production of LKADH. To the best of our knowledge, we could not find any *in silico* studies for secretory production of LKADH.

## MATERIALS AND METHODS

**Dataset retrieval: **“Signal Peptide Website” (http://www.signalpeptide.de/) was employed to retrieve 41 appropriate SPs. SPs were chosen according to several criteria. Selected SPs were marked as confirmed in the mentioned database and belonged to bacterial secretory proteins. The collected data were validated using the “UniProt” server (http://www.uniprot.org/) according to the experimental evidences. The amino acid sequence of LKADH was retrieved from the UniProt (Table 1).

**Table 1:** Amino acid sequences of the signal peptides

**Accession** **No. (Uniprot)**	**Signal Peptide**	**Protein name**	**Source**	**Amino Acid Sequence**
P00634	PPB_ECOLI	Alkaline phosphatase	*Escherichia coli (strain K12)*	**MKQS**TIALALLPLLFTPVTKA
P02932	PHOE_ECOLI	Outer membrane pore protein E	*Escherichia coli (strain K12)*	**MKKS**TLALVVMGIVASASVQA
P0A910	OMPA_ECOLI	Outer membrane protein A	*Escherichia coli (strain K12)*	**MKK**TAIAIAVALAGFATVAQA
P02931	OMPF_ECOLI	Outer membrane protein F	*Escherichia coli (strain K12)*	**MMKR**NILAVIVPALLVAGTANA
P09169	OMPT_ECOLI	Protease 7	*Escherichia coli (strain K12)*	**MRAK**LLGIVLTTPIAISSFA
P06996	OMPC_ECOLI	Outer membrane protein C	*Escherichia coli (strain K12)*	**MKVK**VLSLLVPALLVAGAANA
P13811	ELBH_ECOLX	Heat-labile enterotoxin B chain	*Escherichia coli*	**MNK**VKFYVLFTALLSSLCAHG
P02943	LAMB_ECOLI	Maltoporin	*Escherichia coli (strain K12)*	**MMITLRK**LPLAVAVAAGVMSAQAMA
P0AEX9	MALE_ECOLI	Maltose-binding periplasmic protein	*Escherichia coli (strain K12)*	**MKIK**TGARILALSALTTMMFSASALA
P0AEG4	DSBA_ECOLI	Thiol:disulfide interchange protein dsbA	*Escherichia coli (strain K12)*	**MKK**IWLALAGLVLAFSASA
P0AEE5	DGAL_ECOLI	D-galactose-binding periplasmic protein	*Escherichia coli (strain K12)*	**MNKK**VLTLSAVMASMLFGAAAHA
P38683	TORT_ECOLI	Periplasmic protein torT	*Escherichia coli (strain K12)*	**MR**VLLFLLLSLFMLPAFS
P0A855	TOLB_ECOLI	Protein tolB	*Escherichia coli (strain K12)*	**MKQ**ALRVAFGFLILWASVLHA
P22542	HSTI_ECOLX	Heat-stable enterotoxin II	*Escherichia coli (strain K12)*	**MKK**NIAFLLASMFVFSIATNAYA
P62593	BLAT_ECOLX	Beta-lactamase TEM	*Escherichia coli*	**MSIQ**HFRVALIPFFAAFCLPVFA
P00805	ASPG2_ECOLI	l-asparaginase 2	*Escherichia coli (strain K12)*	**MEFFKK**TALAALVMGFSGAALA
A2TJI4	CEXE_ECOLX	Protein cexE	*Escherichia coli*	**MKK**YILGVILAMGSLSAIA
P05458	PTRA_ECOLI	Protease 3	*Escherichia coli (strain K12)*	**MPR**STWFKALLLLVALWAPLSQA
P45523	FKBA_ECOLI	FKBP-type peptidyl-prolyl cis–trans isomerase	*Escherichia coli*	**MKSLFK**VTLLATTMAVALHAPITFA
P69776	LPP_ECOLI	Major outer membrane lipoprotein	*Escherichia coli (strain K12)*	**MKATK**LVLGAVILGSTLLAG
P31550	THIB_ECOLI	Thiamine-binding periplasmic protein	*Escherichia coli (strain K12)*	**MLKK**CLPLLLLCTAPVFA
Q47537	TAUA_ECOLI	Taurine-binding periplasmic protein	*Escherichia coli (strain K12)*	**MAISSRN**TLLAALAFIAFQAQA
P23857	PSPE_ECOLI	Phage shock protein E	*Escherichia coli (strain K12)*	**MFKK**GLLALALVFSLPVFA
P07102	PPA_ECOLI	Periplasmic appA protein	*Escherichia coli (strain K12)*	**MK**AILIPFLSLLIPLTPQSAFA
P34210	OMPP_ECOLI	Outer membrane protease ompP	*Escherichia coli (strain K12)*	**MQTK**LLAIMLAAPVVFSSQEASA
P24093	DRAA_ECOLX	Dr hemagglutinin structural subunit	*Escherichia coli (strain K12)*	**MKK**LAIMAAASMVFAVSSAHA
P0A915	OMPW_ECOLI	Outer membrane protein W	*Escherichia coli (strain K12)*	**MKK**LTVAALAVTTLLSGSAFA
P0AFI5	PBP7_ECOLI	D-alanyl-D-alanine endopeptidase	*Escherichia coli (strain K12)*	**MPK**FRVSLFSLALMLAVPFAPQAVA
P33590	NIKA_ECOLI	Nickel-binding periplasmic protein	*Escherichia coli (strain K12)*	**MLSTLRR**TLFALLACASFIVHA
P0ADV1	LPTA_ECOLI	Lipopolysaccharide export system protein lptA	*Escherichia coli (strain K12)*	**MKFK**TNKLSLNLVLASSLLAASIPAFA
P16397	SUBF_BACSU	Bacillopeptidase F	*Bacillus subtilis*	**MRKK**TKNRLISSVLSTVVISSLLFPGAAGA
Q02113	CWBA_BACSU	Amidase enhancer	*Bacillus subtilis*	**MKSCK**QLIVCSLAAILLLIPSVSFA
P34957	QOX2_BACSU	Quinol oxidase subunit 2	*Bacillus subtilis*	**M**VIFLFRALKPLLVLALLTVVFVLGG
O07921	CHIS_BACSU	Chitosanase	*Bacillus subtilis*	**MKISMQKADFWKK**AAISLLVFTMFFTLMMSETVFA
P21130	SACB_BACAM	Levansucrase	*Bacillus amyloliquefaciens*	**MNIKKIVK**QATVLTFTTALLAGGATQAFA
P39824	BLAC_BACSU	Beta-lactamase	*Bacillus subtilis*	**MKLKTKASIK**FGICVGLLCLSITGFTPFFNSTHAEA
P07980	GUB_BACAM	Beta-glucanase	*Bacillus amyloliquefaciens*	**MKR**VLLILVTGLFMSLCGITSSVSA
P31797	CDGT_BACST	Cyclomaltodextrin glucanotransferase	*Bacillus stearothermophilus*	**MRRW**LSLVLSMSFVFSAIFIVSDTQKVTVEA
P06874	THER_BACST	Thermolysin	*Bacillus stearothermophilus*	**MNKR**AMLGAIGLAFGLLAAPIGASA
P00808	BLAC_BACLI	Beta-lactamase	*Bacillus licheniformis*	**MK**LWFSTLKLKKAAAVLLFSCVALAG
P06278	AMY_BACLI	Alpha amylase	*Bacillus licheniformis*	**MKQQKRLYAR**LLTLLFALIFLLPHSAAAA

The amino acids in the n-region are boldfaced and the underlined amino acids shows the c-region.

**Prediction of signal peptides presence and their cleavage site location: **“SignalP 4.1” (http://www.cbs.dtu.dk/services/SignalP/) is an online web server that distinguishes three regions of SPs and their presence probability for target protein based on artificial neural networks (ANNs). SignalP was upgraded to version 4.1 in 2012 with the advent of a cut-off value that was named D-score. D-score is used for the final decision about SPs presence in N-terminus of input amino acid sequences. In this study, if a sequence had a D-score higher than 0.57 was considered as SP. SignalP results for each amino acid sequence are made of three scores based on the neural networks. SPs cleavage sites are determined using C-score (raw cleavage site score). S-score (signal peptide score) distinguishes the sequence of SPs from the target protein sequence and proteins without SPs. Y-score (combined cleavage site score) is the geometric average of the C-score and the slope of the S-score, which differentiate cleavage site prediction better than the raw C score alone [13]. SignalP results were rechecked by “Philius” (http://www.yeastrc.org/philius/). A sequence with type confidence more than 0.5 was considered as signal peptide.

**Evaluation of signal peptides physico-chemical properties and solubility: **“ProtParam” is an online server at (http://web.expasy.org/), which was employed to predict the different physico-chemical properties of the SPs, including amino acid composition, molecular weight, theoretical pI (isoelectric point), positively charged residues, instability index, aliphatic index, and grand average of hydropathicity (GRAVY). ProtParam evaluates these features based on a protein sequence [14]. SPs instability index separately and connected to LKADH was evaluated. The solubility of SPs and LKADH fusions were predicted using “Protein-sol” online software. Protein–Sol (http://protein-sol.manchester.ac.uk) is a free online web server. Protein–Sol gives a predicted scaled solubility in the 0-1 range to interpret results easily [15]. Unstable fusion proteins and insoluble ones were removed in the next step.

***In silico*** **prediction of signal peptides secretion pathway and subcellular localization: **“PRED-TAT” online server (http://www.compgen.org/tools/PRED-TAT) was used to predict SPs connected LKADH fusions secretion pathway. PRED-TAT differentiates Sec from Tat targeting SPs and predicts their cleavage sites by providing a reliability score in the 0-1 range. The prediction method of the aforementioned server is dependent on Hidden Markov Models (HMMs) and has a standard appropriate architecture for both Sec and Tat SPs [16]. Sub-cellular localization of SPs connected LKADH fusions was evaluated using “ProtCompB” online server (http://www.softberry.com). Its prediction of the localized fusion was based on neural networks, containing the last localization database of homologous proteins. The average accuracy of “ProtCompB” is between 86-100% [17, 18].

## RESULTS

**Evaluation of signal peptides three regions and probability: **The amino acid length for all selected SPs in the n-, h- and c-regions were 2-10, 10-20, and 3-9, respectively. The most critical parameter to identify SPs presence was D-score. If a SP had a D-score higher than 0.57, it was considered as appropriate SP for the target protein. The *in silico* analysis indicated that the highest D-score belonged to CWBA_BACSU (0.916). QOX2_BACSU (0.217), CHIS_BACSU (0.313), LPP_ECOLI (0.342), BLAC_BACSU (0.377), CDGT_BACST (0.388), BLAT_ECOLX (0.443), GUB_BACAM (0.463), TOLB_ECOLI (0.471), THER_BACST (0.510) and OMPT_ECOLI (0.524) were not suitable SPs for the excretion of LKADH protein, since they had D-scores below 0.57 (Table 2). These signal peptides were removed in the next step. 

**Table 2:** *In silico* prediction of signal peptides for LKADH

**Protein Name**	**SignalP analysis**									**Philius analysis**
	**n-region**	**h-region**	**c-region**	**Cleavage Site**	**C-score**	**Y-score**	**S-score**	**S-mean**	**D-score**	**Type confidence**
PPB_ECOLI	1-4(4)	5-15(11)	16-21(6)	TKA-MT (21,22)	0.393	0.506	0.868	0.700	0.597	0.99
PHOE_ECOLI	1-4(4)	5-17(13)	18-21(4)	VQA-MT (21,22)	0.688	0.738	0.901	0.804	0.769	0.99
OMPA_ECOLI	1-3(3)	4-16(13)	17-21(5)	AQA-MT (21,22)	0.751	0.794	0.954	0.861	0.825	0.99
OMPF_ECOLI	1-4(4)	5-18(14)	19-22(4)	ANA-MT (22,23)	0.805	0.817	0.937	0.864	0.839	0.99
OMPT_ECOLI	*	-	-	-	0.307	0.427	0.808	0.633	0.524	0.98
OMPC_ECOLI	1-4(4)	5-16(10)	17-20(4)	ANA-MT (21,22)	0.784	0.829	0.956	0.901	0.863	0.99
ELBH_ECOLX	1-3(3)	4-17(14)	18-21(4)	AHG-MT (21,22)	0.750	0.734	0.912	0.746	0.740	0.99
LAMB_ECOLI	1-7(7)	8-19(12)	20-25(6)	AMA-MT (25,26)	0.709	0.760	0.964	0.877	0.815	0.96
MALE_ECOLI	1-4(4)	5-19(15)	20-26(7)	ALA-MT (26,27)	0.630	0.754	0.981	0.923	0.834	0.98
DSBA_ECOLI	1-3(3)	4-14(11)	15-19(5)	ASA-MT (19,20)	0.565	0.723	0.959	0.922	0.816	0.99
DGAL_ECOLI	1-4(4)	5-16(10)	17-23(7)	AHA-MT (23,24)	0.690	0.781	0.967	0.910	0.841	0.98
TORT_ECOLI	1-2(2)	3-14(12)	15-18(4)	AFS-MT (18,19)	0.517	0.694	0.957	0.928	0.804	0.99
TOLB_ECOLI	*	-	-	-	0.457	0.469	0.607	0.474	0.471	0.99
HSTI_ECOLX	1-3(3)	4-18(15)	19-23(5)	AYA-MT (23, 24)	0.820	0.824	0.956	0.877	0.849	0.97
BLAT_ECOLX	*	-	-	-	0.536	0.454	0.549	0.425	0.443	0.99
ASPG2_ECOLI	1-6(6)	7-18(12)	19-22(4)	ALA-MT (22,23)	0.805	0.773	0.941	0.770	0.772	0.99
CEXE_ECOLX	1-3(3)	4-14(11)	15-19(5)	AIA-MT (19,20)	0.639	0.712	0.943	0.799	0.753	0.97
PTRA_ECOLI	1-6(6)	7-20(14)	21-25(5)	SQA-MT (23,24)	0.753	0.833	0.964	0.920	0.874	0.99
FKBA_ECOLI	1-6(6)	7-20(14)	21-25(5)	TFA-MT (25,26)	0.522	0.607	0.957	0.819	0.706	0.99
LPP_ECOLI	*	-	-	-	0.148	0.251	0.689	0.497	0.342	0.99
THIB_ECOLI	1-4(4)	5-14(10)	15-18(4)	VFA-MT (18,19)	0.515	0.656	0.919	0.845	0.744	0.99
TAUA_ECOLI	1-7(7)	8-17(10)	18-22(5)	AQA-MT (22,23)	0.775	0.741	0.919	0.775	0.757	0.99
PSPE_ECOLI	1-4(4)	5-15(11)	16-19(4)	VFA-MT (19,20)	0.850	0.858	0.952	0.876	0.866	0.99
PPA_ECOLI	1-2(2)	3-13(11)	14-22(9)	AFA-MT (22, 23)	0.728	0.734	0.896	0.791	0.761	0.99
OMPP_ECOLI	1-4(4)	5-15(11)	16-23(8)	ASA-MT (23,24)	0.517	0.577	0.853	0.724	0.646	0.95
DRAA_ECOLX	1-3(3)	4-13(10)	14-21(8)	AHA-MT (21,22)	0.622	0.735	0.946	0.895	0.810	0.99
OMPW_ECOLI	1-3(3)	4-13(10)	14-21(8)	AFA-MT (21,22)	0.759	0.816	0.949	0.889	0.850	0.99
PBP7_ECOLI	1-3(3)	4-17(14)	18-25(8)	AVA-MT (25,26)	0.589	0.695	0.972	0.886	0.785	0.99
NIKA_ECOLI	1-7(7)	8-19(12)	20-22(3)	VHA-MT (22,23)	0.775	0.804	0.925	0.861	0.831	0.99
LPTA_ECOLI	1-4(4)	5-23(19)	24-27(4)	AFA-MT (27,28)	0.828	0.846	0.977	0.912	0.877	0.99
SUBF_BACSU	1-4(4)	5-23(19)	24-30(7)	AGA-MT (30,31)	0.581	0.669	0.955	0.859	0.758	0.99
CWBA_BACSU	1-5(5)	6-19(18)	20-25(6)	SFA-MT (25,26)	0.853	0.894	0.976	0.940	0.916	0.99
QOX2_BACSU	*	-	-	-	0.136	0.170	0.372	0.296	0.217	0.99
CHIS_BACSU	*	-	-	-	0.357	0.331	0.536	0.283	0.313	0.86
SACB_BACAM	1-8(8)	9-24(16)	25-29(5)	AFA-MT (29,30)	0.558	0.596	0.951	0.772	0.679	0.97
BLAC_BACSU	*	-	-	-	0.274	0.302	0.752	0.504	0.377	0.95
GUB_BACAM	*	-	-	-	0.307	0.398	0.715	0.575	0.463	0.98
CDGT_BACST	*	- -	- -	-	0.130	0.280	0.706	0.572	0.388	0.99
THER_BACST	*	-	-	-	0.257	0.400	0.813	0.699	0.510	0.98
BLAC_BACLI	1-2(2)	3-22(20)	23-26(4)	ALA-GM (25,26)	0.756	0.824	0.965	0.914	0.866	0.99
AMY_BACLI	1- 10(10)	11-22(12)	23-29(7)	AAA-AM (28,29)	0.452	0.590	0.889	0.665	0.618	0.99

SignalP 4.1 outputs includes several different scores. The C-score and S-score were used for determination of cleavage sites and signal peptides positions, respectively. Y-score indicates the geometric average between the C-score and a smoothed derivative of the S-score. S-mean is arithmetic average of the S-score from the beginning to position where the Y-score is the max. D-score is the mean of the S-mean and Y-max which determines secretory and non-secretory proteins with cut-off value of 0.5. Sequences with D-score > 0.5 are considered as signal peptide.

**Physicochemical properties and solubility of signal peptides: **Various physico-chemical features of the SPs are shown in Table 3. We chose the SPs length in the range of 18-30. The net positive charge of n-region was 0 for OMPP_ECOLI, 1 for TORT_ECOLI, BLAT_ECOLX, ASPG2_ECOLI, TAUA_ECOLI and PPA_ECOLI, 3 for MALE_ECOLI, LPTA_ECOLI, SACB_BACAM and BLAC_BACSU, 4 for BLAC_BACLI and AMY_BACLI, 5 for SUBF_BACSU and 2 for the other 20 SPs. The grand average of hydropathy (GRAVY) is defined as the sum of hydropathy of amino acids and implemented for total hydropathy comparison. [14]. SUBF_BACSU (0.497) and TORT_ECOLI (2.061) had the lowest and highest GRAVYs. The hydrophobicity value is indicated, using the aliphatic index, related to the aliphatic amino acids (i.e., alanine, valine, isoleucine, and leucine) composition of a protein sequence. [14]. QOX2_BACSU (198.46) had the highest aliphatic index, unlike the lowest one which belonged to BLAC_BACSU (92.22). The instability of the signal peptides alone and in connection with LKADH protein were predicted by the instability index. Having an instability index below 40 indicated the stability of a protein and vice versa. Based on our results, BLAC_BACSU (15.03) in connection with LKADH was the most stable fusion protein.

**Table 3:** Prediction of signal peptides physico-chemical properties

**Signal Peptides**	**Amino Acid** **Length**	**MW**	**pI**	**Net Positive Charge**	**Aliphatic Index**	**GRAVY**	**Instability Index** **Without protein**	**Instability index with protein**	**Solubility** **(Probability)**
PPB_ECOLI	21	2256.82	10.00	2	139.52	0.971	Unstable (56.02)	Unstable (19.61)	Soluble(0.661)
PHOE_ECOLI	21	2104.59	10.00	2	130.00	1.195	Stable (1.44)	Stable (15.42)	Soluble(0.688)
OMPA_ECOLI	21	2046.50	10.00	2	121.43	1.295	Stable (9.52)	Stable (16.04)	Soluble(0.697)
OMPF_ECOLI	22	2266.83	11.00	2	150.91	1.259	Unstable (67.18)	Stable (20.64)	Soluble(0.678)
OMPT_ECOLI	20	2102.61	11.00	2	146.50	1.290	Stable (2.62)	Stable (15.55)	Soluble(0.672)
OMPC_ECOLI	21	2078.63	10.00	2	171.90	1.552	Stable (14.37)	Stable (16.41)	Soluble(0.695)
ELBH_ECOLX	21	2358.84	9.10	2	111.43	0.695	Stable (26.85)	Stable (17.37)	Soluble(0.608)
LAMB_ECOLI	25	2545.22	11.00	2	125.20	1.332	Unstable (42.97)	Stable (18.96)	Soluble(0.668)
MALE_ECOLI	26	2698.34	11.17	3	113.08	1.012	Stable (2.85)	Stable (15.30)	Soluble(0.657)
DSBA_ECOLI	19	1990.48	10.00	2	144.21	1.416	Stable (11.50)	Stable (16.22)	Soluble(0.662)
DGAL_ECOLI	23	2362.89	10.00	2	102.17	0.952	Stable (14.15)	Stable (16.38)	Soluble(0.668)
TORT_ECOLI	18	2111.72	9.50	1	173.33	2.061	Stable (26.66)	Stable (17.25)	Soluble(0.640)
TOLB_ECOLI	21	2371.92	11.00	2	139.52	1.219	Unstable (43.26)	Stable (18.63)	Soluble(0.618)
HSTI_ECOLX	23	2552.09	9.70	2	102.17	1.026	Stable (32.43)	Stable (17.91)	Soluble(0.608)
BLAT_ECOLX	23	2626.22	8.02	1	110.43	1.539	Unstable (56.40)	Stable (19.91)	Soluble(0.573)
ASPG2_ECOLI	22	2274.76	8.35	1	93.64	1.136	Stable (-1.15)	Stable (15.16)	Soluble(0.683)
CEXE_ECOLX	19	1979.51	9.70	2	154.21	1.411	Stable (29.75)	Stable (17.50)	Soluble(0.678)
PTRA_ECOLI	23	2613.20	11.00	2	131.74	0.857	Unstable (51.93)	Stable (19.54)	Soluble(0.604)
FKBA_ECOLI	25	2676.31	10.00	2	121.20	1.212	Stable (14.37)	Stable (16.38)	Soluble(0.649)
LPP_ECOLI	20	1956.46	10.00	2	161.00	1.400	Stable (10.64)	Stable (16.14)	Soluble(0.707)
THIB_ECOLI	18	1974.60	8.89	2	157.22	1.589	Unstable (65.64)	Stable (19.85)	Soluble(0.646)
TAUA_ECOLI	22	2308.72	9.50	1	120.45	1.055	Stable(34.41)	Stable (18.01)	Soluble(0.659)
PSPE_ECOLI	19	2065.63	10.00	2	148.95	1.711	Stable (17.37)	Stable (16.64)	Soluble(0.643)
PPA_ECOLI	22	2384.99	8.50	1	155.45	1.405	Unstable (53.16)	Stable (19.52)	Soluble(0.645)
OMPP_ECOLI	23	2406.88	5.75	0	114.78	0.904	Unstable (44.47)	Stable (18.91)	Soluble(0.697)
DRAA_ECOLX	21	2135.63	10.00	2	98.10	1.162	Stable (16.49)	Stable (16.57)	Soluble(0.674)
OMPW_ECOLI	21	2093.55	10.00	2	125.71	1.210	Stable (1.44)	Stable (15.42)	Soluble(0.694)
PBP7_ECOLI	25	2705.36	11.00	2	117.20	1.228	Unstable (57.99)	Stable (20.32)	Soluble(0.611)
NIKA_ECOLI	22	2434.99	10.35	2	137.73	1.350	Unstable (60.45)	Stable (20.10)	Soluble(0.634)
LPTA_ECOLI	27	2849.47	10.30	3	130.37	0.881	Stable (17.32)	Stable (16.65)	Soluble(0.628)
SUBF_BACSU	30	3145.80	12.02	5	117.00	0.497	Stable (20.75)	Stable (17.02)	Soluble(0.634)
CWBA_BACSU	25	2649.35	8.89	2	160.00	1.596	Unstable (42.97)	Stable (18.96)	Soluble(0.634)
QOX2_BACSU	26	2843.68	11.00	2	198.46	2.242	Stable (8.20)	Stable (15.80)	Soluble(0.646)
CHIS_BACSU	35	4094.03	9.52	2	83.71	0.800	Stable (17.79)	Stable (16.73)	Soluble(0.577)
SACB_BACAM	29	3008.61	10.30	3	107.93	0.710	Stable (26.18)	Stable (17.57)	Soluble(0.642)
BLAC_BACSU	36	3875.66	9.30	3	92.22	0.628	Stable (4.18)	Stable (15.03)	Soluble(0.575)
GUB_BACAM	25	2640.30	9.50	2	148.00	1.508	Unstable (45.44)	Stable (19.18)	Soluble(0.657)
CDGT_BACST	31	3562.25	8.50	1	116.13	0.887	Unstable (46.86)	Stable (19.90)	Soluble(0.632)
THER_BACST	25	2414.95	11.00	2	121.60	1.100	Stable (33.02)	Stable (18.06)	Soluble(0.678)
BLAC_BACLI	26	2810.54	10.04	4	131.54	1.192	Stable (15.30)	Stable (16.46)	Soluble(0.607)
AMY_BACLI	29	3299.07	11.10	4	141.72	0.752	Unstable (50.51)	Stable (20.08)	Soluble(0.577)

MW (molecular weight), pI (isoelectric point), Instability index, GRAVY (grand average of hydropathicity). Proteins with instability index more than 40 were considered as unstable.

**Prediction of secretion pathway and sub-cellular localization: **The results of PRED-TAT web server revealed that all the remaining stable and soluble SPs belonged to the Sec pathway, except QOX2_BACSU that targets the protein to transmembrane segment. These SPs can translocate fused LKADH to different compartments. ProtCompB server sub-cellular localization evaluation, indicating that amongst SPs in this step, LPTA_ECOLI and SACB_BACAM can localize LKADH in periplasmic space, SUBF_BACSU, CHIS_BACSU, CDGT_BACST and AMY_BACLI can translocate this heterologous protein into extracellular space, and other SPs can direct this heterologous protein into the cytoplasm (Table 4).

**Table 4:** Evaluation of secretion pathways and sub-cellular location of SPs

**Signal peptides**	**Secretion pathway**	**Reliability** **Score (%)**	**Cytoplasmic**	**Membrane**	**Secreted** **(extracellular)**	**Periplasmic**	**Final prediction site**
PHOE_ECOLI	Sec	0.995	9.91	0.03	0.00	00.06	Cytoplasmic
OMPA_ECOLI	Sec	0.999	9.82	0.09	0.03	0.06	Cytoplasmic
OMPF_ECOLI	Sec	0.998	9.90	0.04	0.00	0.06	Cytoplasmic
OMPT_ECOLI	Sec	0.991	9.93	0.02	0.00	0.05	Cytoplasmic
OMPC_ECOLI	Sec	0.990	9.84	0.09	0.00	0.07	Cytoplasmic
ELBH_ECOLX	Sec	0.985	9.80	0.12	0.02	0.06	Cytoplasmic
LAMB_ECOLI	Sec	0.998	9.92	0.02	0.00	0.06	Cytoplasmic
MALE_ECOLI	Sec	0.997	9.92	0.06	0.00	0.02	Cytoplasmic
DSBA_ECOLI	Sec	0.994	9.83	0.11	0.00	0.05	Cytoplasmic
DGAL_ECOLI	Sec	0.999	9.83	0.11	0.00	0.06	Cytoplasmic
TORT_ECOLI	Sec	0.990	9.96	0.02	0.00	0.03	Cytoplasmic
TOLB_ECOLI	Sec	0.991	9.85	0.11	0.00	0.04	Cytoplasmic
HSTI_ECOLX	Sec	0.999	9.62	0.10	0.24	0.04	Cytoplasmic
BLAT_ECOLX	Sec	0.996	9.89	0.00	0.07	0.03	Cytoplasmic
ASPG2_ECOLI	Sec	0.999	9.80	0.13	0.01	0.06	Cytoplasmic
CEXE_ECOLX	Sec	0.993	9.82	0.06	0.07	0.05	Cytoplasmic
PTRA_ECOLI	Sec	0.995	9.82	0.12	0.00	0.05	Cytoplasmic
FKBA_ECOLI	Sec	0.992	9.81	0.15	0.00	0.04	Cytoplasmic
LPP_ECOLI	Sec	0.968	9.89	0.05	0.00	0.06	Cytoplasmic
THIB_ECOLI	Sec	0.997	9.86	0.09	0.00	0.06	Cytoplasmic
TAUA_ECOLI	Sec	0.998	9.90	0.04	0.00	0.06	Cytoplasmic
PSPE_ECOLI	Sec	0.999	9.72	0.10	0.13	0.05	Cytoplasmic
PPA_ECOLI	Sec	1.000	9.82	0.12	0.00	0.06	Cytoplasmic
OMPP_ECOLI	Sec	0.997	9.93	0.02	0.00	0.05	Cytoplasmic
DRAA_ECOLX	Sec	1.000	9.85	0.08	0.00	0.06	Cytoplasmic
OMPW_ECOLI	Sec	1.000	9.82	0.10	0.02	0.06	Cytoplasmic
PBP7_ECOLI	Sec	0.997	9.83	0.12	0.00	0.05	Cytoplasmic
NIKA_ECOLI	Sec	0.996	9.88	0.07	0.00	0.06	Cytoplasmic
LPTA_ECOLI	Sec	1.000	0.00	0.60	1.12	8.28	Periplasmic
SUBF_BACSU	Sec	0.996	2.22	1.85	5.77	0.16	Secreted
CWBA_BACSU	Sec	0.999	9.94	0.00	0.02	0.04	Cytoplasmic
QOX2_BACSU	TM segment	0.788	9.95	0.00	0.02	0.03	Cytoplasmic
CHIS_BACSU	Sec	0.975	2.20	0.84	6.77	0.19	Secreted
SACB_BACAM	Sec	0.999	1.04	0.00	0.00	8.96	Periplasmic
BLAC_BACSU	Sec	0.995	2.36	0.00	2.41	5.23	membrane bound Periplasmic
GUB_BACAM	Sec	0.998	9.93	0.00	0.03	0.04	Cytoplasmic
CDGT_BACST	Sec	0.958	1.37	1.69	6.94	0.00	Secreted
THER_BACST	Sec	0.997	9.74	0.03	0.19	0.04	Cytoplasmic
BLAC_BACLI	Sec	0.992	9.91	0.03	0.00	0.06	Cytoplasmic
AMY_BACLI	Sec	0.999	0.00	1.57	8.43	0.00	Secreted

## DISCUSSION

Overexpression of recombinant proteins in the intracellular space of *E. coli *is usually accompanied with high inclusion body aggregation; hence, it is essential to launch a method for periplasmic or extracellular secretion of proteins [9]. Sec, SRP and TAT are some of protein secretion pathways, which are recruited by prokaryotes. The role of these pathways is to direct proteins into periplasmic space according to their SPs. Thus, choosing a proper SP is a critical step in designing secretory recombinant proteins [11]. The new era in medical and biology has begun with the advent of some other sciences, such as computational biology and bioinformatics. The advantages of using bioinformatics program before launching an experimental study are reducing the costs and increasing the accuracy and validity of the experimental researches [19]. 

There are many of these bioinformatics online web tools, which can be used to find suitable SPs. A parameter that determines a peptide as a SP is D-score of the signalP server; hence, D-score is used to sort all SPs in the first step. According to D-scores (Table 2), 31 out of 41 selected SPs were identified as SPs for LKADH, but more features were needed to evaluate a suitable SP. 

Some important physico-chemical characteristics of SPs including instability index, GRAVY, net positive charge, h-region length has to be considered for effective protein secretion. A crucial region in a SP is n-region that can confer the ability for translocation to the desired secretory protein. The existence of one or more basic residues causes the n-region to be positively charged. The positive charges facilitate the interaction between SPs and phospholipids, which helps protein to translocate through the membrane [20]. Therefore, any substitution that changes the basic residues with neutral or acidic ones in the signal peptide sequences can reduce the rate of protein synthesis and their secretion [21]. The results of the present study showed a range of 0-5 for positive charges of all SPs. As all the selected SPs are in a suitable range of positively charged residues for n-region, it is also necessary to consider other characteristics for selecting a suitable SP. The h-region is another key region of a SP that its hydrophobic feature has an essential role in membrane targeting and extracellular secretion of proteins [22]. Therefore, hydrophobicity is the most crucial factor for the activity of this region, and the length of h-region is also a determinant of hydrophobicity. Consequently, increasing the length of h-region can raise the level of hydrophobicity, helping to promote the protein secretion rate. Aliphatic index and GRAVY are two major parameters that determine the hydrophobicity, and any increase in these parameters can lead to elevated hydrophobicity [18, 22]. As shown in Table 3, all 31 evaluated SPs in this step of the study had high aliphatic indexes, and their hydrophobicity is suitable for secretion. During protein transportation into the extracellular space, signal peptidases cleave the signal peptide sequence in the cleavage site and produce a mature protein product. The cleavage site located in the c-region often has less hydrophobicity, and it includes a signal sequence that is recognized by the signal peptidase. According to the -1, -3 rule a residue with a small neutral side chain like alanine, serine and glycine should be located at -1 and -3 positions. Hence, an Ala-X-Ala box sequence which forms, can be identified and cleaved by signal peptidase [23]. As shown in Table 1, alanine is the most common residue found at the -1 and -3 positions, and the others are approximately similar to AXA box. 

Two common translocating pathways in both gram-negative and -positive bacteria that trigger proteins to the extracellular space, are Sec and Tat [24]. In *E. coli*, about 50% of all total proteins are excreted, with more than 90% secreted via the Sec pathway. Using the Sec pathway, the unfolded proteins can translocate across the membrane and target the extracellular space, either via co-translational (SRP pathway) or post-translational. On the other hand, fully folded proteins get out of the cytoplasm by Tat pathway, a process that uses Tat translocation complex. Folding of proteins in the cytoplasm can result in their aggregation and degradation due to cellular proteases; thus, it seems that Sec and SRP pathways are more suitable for the secretory production of proteins than Tat [25-27]. As indicated in Table 4, all SPs in this step were specific for the Sec pathway with reliability scores of more than 0.9. For this reason, based on the analysis none of them were omitted, since we required sub-cellular localization analysis. 

At the end, it was determined that amongst 30 stable and soluble LKADH fused SPs, directed toward Sec pathway, 26 SPs were able to translocated to the cytoplasm, and only 4 translocated to LKADH into the periplasmic and extracellular space. 

 As far as we know, this is the first study aiming to investigate suitable SPs in fusion with LKADH by analyzing their potential effects on the secretion of this protein. It is logical that selecting a suitable and accurate SP for a given protein can reduce the cost and time for production and purification processes of recombinant proteins. This study evaluated 41 diﬀerent SPs in order to select the most applicable ones for secreting the recombinant LKADH protein out of *E. coli *host. The results of this work indicated that LPTA_ECOLI, SUBF_BACSU, CHIS_BACSU, SACB_BACAM, CDGT_BACST and AMY_BACLI SPs could be theoretically considered as suitable candidates for the LKADH secretion. However, further experimental investigations should be carried out to validate these results.
